# Proceedings of Réanimation 2018, the French Intensive Care Society International Congress: physiotherapists’ communications

**DOI:** 10.1186/s13613-018-0390-x

**Published:** 2018-05-02

## Co-41

### Research and virtualization in chest physiotherapy (ANR-16-CE19-0014)

#### Dominique Pelca^1^, Jean-Claude Jeulin^2^, Christian Fausser^3^

##### ^1^Pierrefitte, France; ^2^Annecy Le Vieux, France; ^3^Alfortville, France

**Correspondence:** Dominique Pelca - mkdompe@gmail.com

*Annals of Intensive Care* 2018, **8(Suppl 2):**Co-41

**Introduction:** Recent research in the areas of the simulation and virtualization have encouraged us, and permits to refine our approach in the framework of chest physiotherapy for newborn infants. The Multidisciplinary Team original as we have incorporated brings together researchers in applied mathematics, physics, biomechanics, mechatronics, fluid mechanics, respiratory physiology and physiotherapists. Here we develop the approach used for the design of the simulator, and the theoretical elements which support.

**Patients and Methods:** This study is based on three separate sub-models- one for the lung, one for the thorax and one to mimic chest physiotherapy manipulations. We build a minimal model, which includes only the core properties we identified as playing an important role in chest physiotherapy. This choice was mainly made in order to be able to interpret correctly the interactions between these core phenomena. It was also a way to limit the computation times of the numerical studies. As a consequence, our model can perform qualitative and comparative predictions, but not quantitative predictions.

**Results:** Our results indicate that manipulations need to overcome secretions threshold in order to be able to mobilize secretions. Our model shows that manipulations main effect is to reduce the hydrodynamic resistance of the airway tree by secretions redistribution in the tree, eventually reaching a distribution that is not anymore affected by the manipulation. This effect means that patient breathing might be instantly improved by the manipulation. The second main effect is mucus expectoration, which is also another mean to improve the status of the patient. Mucus expectoration predicted by our model is nonnegligible only for pressures high enough, and also, in the case of high frequency chest wall oscillation (HFCWO), for frequencies high enough. These conclusions, essentially mechanistic, do not depend qualitatively on the initial mucus distribution.

**Conclusion:** This collaboration allows us today to confront our practice to the theory, test our assumptions, in coherence with the scientific data updated. Finally, we proposed two numbers to measure the efficiency of the manipulations- the Shrek number and the comfort number. They are both performing well in the case of our idealized manipulations. They need however to be validated with clinical studies. The first mathematical and digital models from this collaboration are encouraging and offer avenues that we can build on as our works move forward.

## Co-37

### Feasibility and safety of early mobilization in critically ill children

#### Damien Moerman^1,2^, Emilien Derycke^1^, Pauline Bednarek^1^, Cheryl Hickmann^1^, Laurent Houtekie^1^, Astrid Haenecour^1^, Thierry Detaille^1^, Stephan Clément de Cléty^1^

##### ^1^Pediatric Intensive Care Unit, Cliniques universitaires Saint-Luc, Brussels, Belgium; ^2^Department of Physical Medicine and Rehabilitation, Cliniques universitaires Saint-Luc, Brussels, Belgium

**Correspondence:** Damien Moerman - damien.moerman@uclouvain.be

*Annals of Intensive Care* 2018, **8(Suppl 2):**Co-37

**Introduction:** Early mobilization of adult patients admitted to the intensive care has become current practice since a few years. Indeed, many studies have reported about the benefits of early mobilization. However, early mobilization in pediatric intensive care is poorly described. The aim of this study was to evaluate the feasibility and the safety of early mobilization in critically ill children (0–2 years) and its impact on comfort scores.

**Patients and Methods:** Patients admitted since 24 to 48 h in the Pediatric Intensive Care Unit were screened to identify eligible children, between 0 and 2 years old, stable from a cardiorespiratory point of view. Early mobilization was performed between 24 and 48 h of admission. Respiratory and hemodynamic data were recorded before mobilization (T0), immediately after the treatment (T1) and after 10 min (T2), 30 min (T3) and 1 h (T4). The Comfort Behavior scale and EDIN scale were used for respectively the intubated and the extubated child in order to evaluate comfort during the mobilization session. The comfort scores were taken before mobilization (T0), immediately after the treatment (T1) and after 10 min (T2). Adverse events such as endotracheal tube or catheter loss were recorded. The institutional research ethics board approved the research protol. Written informed consent was obtained from parents or legal guardians. Statistical analysis was performed using SPSS, version 24 (IBM). To compare respiratory and hemodynamic parameters, repeated measures ANOVA test or Friedman Test was used. The same tests were used to compare the series of comfort scores. Statistical significance was fixed at p < 0.05.

**Results:** From September 2016 untill February 2017, 237 children were screened, 65 were eligible, 20 were enrolled and mobilized. The heart rate, respiratory rate, systolic and diastolic blood pressure, oxygen saturation and comfort scores showed no significant change before and after mobilization. Four sessions of mobilization were stopped because of important agitation of the patient with at T1 change in respiratory and hemodynamic values as well as a change in comfort scores. No adverse events were reported.

**Conclusion:** Mobilization is safe and probably feasible in children aged 0–2 years providing physiotherapists can adapt to the behaviour of the child and stop in case of poor tolerance.

## Co-36

### Dup-Reyg- A new system to increase the Fraction of Inspired Oxygen (FiO2) with a Boussignac system

#### Frédéric Duprez^1^, Gregory Cuvelier^2^, Titus Ebogo^3^, Jean-Marie Jacques^3^, Sharam Mashayekhi^3^, Gregory Reychler^4^

##### ^1^ICU, Epicura, Hornu | België Belgium, Belgium; ^2^Condorcet School, Tournai, Belgium; ^3^Emergency Care, Epicura, Hornu, Belgium; ^4^Pneumology, IREC Saint Luc UCL, Bruxelles, Belgium

**Correspondence:** Frédéric Duprez - dtamedical@hotmail.com

*Annals of Intensive Care* 2018, **8(Suppl 2):**Co-36

**Introduction:** Boussignac system (BS) administers oxygen and generates a continuous positive airway pressure (cpap). With the BS, fraction of inspired oxygen (FiO2) value is approximatively equal to the ratio between O2 flow and inspiratory flow (IF). In some cases, FiO2 decreases due to IF increase. To limit the FiO2 decrease during IF increase, we developed a new system- The Dup-Reyg system. The aim of this study was to test the Dup-Reyg system connected to a BS during IF increases.

**Patients and Methods:** The study was conducted on bench with a BS connected to a two-compartment adult lung model (Dual Test Lung^®^- DTL) controlled by a Maquet Servo I^®^ventilator. Three minute ventilations (MV 10 20 and 30 L min) with a Ti Ttot = 0.33 were investigated. FiO2 and MV measurements were made using an iWorx^®^ GA207 gas analyzer. Three Positive End Expiratory Pressure (PEEP) were analyzed- 3, 5 and 10 cm H2O. The BS was supplied with an O2 flow. In order to increases the FiO2 during IF increases, we have evaluated the impact of the addition of the Dup-Reyg system (two corrugated tubing ISO 22, length- 18 cm for each Trunk) connected to expiratory way of BS. A Friedman test followed by a Holm-Sidak method were used to compare data.

**Fig. 1:**
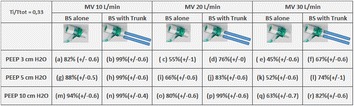
Comparisson of Boussignac system (BS) alone, versus with Trunk

**Results:** Means are expressed with their SD (Fig. 1). No statistical differences (p > .05) were found between (a-j j-r h-p h-n b-h i-f a-r b-n b-p n-p) (Fig. 1). Higher the MV, lower the FiO2 (p < 0.05). Moreover, higher the PEEP, higher the FiO2 (p < 0.05). The addition of the Dup-Reyg system at the entry of BS increases FiO2 (p < 0.05). The impact of the Dup-Reyg addition is more important with high MV. In our study, FiO2’s absolute difference observed were equal from 5 to 22%.

**Conclusion:** The addition of a Dup-Reyg system to the expiratory way of a BS increases FiO2 significantly. This system could be implemented for the purpose of increasing the FiO2 when the IF is high and or when the oxygen flow delivery is limited (ambulance, disaster situations, war zone etc.). However, complementary studies must be carried out in order to verify its clinical application.

## Co-39

### NEMS protocol for Swallowing Disorders patients in ICU

#### Carlos Diaz Lopez^1^, Maria Bonarek^1^, Daniel Ferreiro Carballal^1^, Stéphane Henriot^1^, Andreia Lopes^1^, Julie Marais^1^, Julie Rodriguez^1^, Pascal Selot^1^, Aymeric Le Neindre^1,2^

##### ^1^Service de rééducation, Hôpital Forcilles, Férolles-Attilly, France; ^2^UMR1231, Université de Bourgogne, Dijon, France

**Correspondence:** Carlos Diaz Lopes - carlosdl90@hotmail.com

*Annals of Intensive Care* 2018, **8(Suppl 2):**Co-39

**Introduction:** Swallowing disorders (SD) are a major concern in Intensive Care Unit (ICU), with a prevalence of 15 to 87% (1). SD may be the cause of malnutrition, dehydratation and pneumonia (2). It may increase the hospitalization length, induce higher reintubation rate and delay tracheotomy removing (3) (4). Atrophy of laryngeal muscles, alertness and movement decreasing (related to the presence of endotracheal tube) and the use of sedative or neuromuscular blocking agents are important risk factors of dysphagia. Neuromuscular electrical stimulation (NEMS) is an interesting tool for the physiotherapist and studies provide promising result to support the use of NEMS associated to usual treatment in ICU patients with SD (5). A literature review highlights the lacks of standardized NEMS protocol for SD management in ICU. Objective—we propose a standardized NEMS protocol for SD patients in ICU for future research protocol.

**Patients and Methods:** The protocol aims to include difficult-to-wean patients after a long ICU hospitalization period and suffering from SD. The presence of SD is revealed by the Food and Oral Intake Scale (FOIS) and clinical examination (food and water test, coughing, choking and voice alteration). FOIS is the main outcome to assess the protocol (6). NEMS is associated to conventional SD treatment- Shaker exercices, thermal-tactil stimulation, effortful swallowing maneuver, laryngeal and lingual exercises and compensatory maneuvers. NEMS is performed with a Cephar Physio4 and small conventional electrodes. Electrodes are placed to supra and infrahyoid muscles and above the superior limit of thyroid notch. Two-phases current is used with frequencies varying between 5 to 80 Hz in three differents periods (warming-up, stimulation and relaxing). The total of the NEMS treatment varies between 16 to 26 min, one session per day until swallowing improvement or patient discharge.

**Results:** NEMS associated to usual therapy may improve the SD management. Literature highlights a decrease of lenght of hospitalisation and a higher FOIS evolution with the use of NEMS. We aim to published this protocol for supporting the use of a standardized NEMS protocol in clinical research.

**Discussion:** Additional studies are required to assess the efficacy of NEMS with a standardized protocol. Moreover, FOIS has limitations to evaluate SD and new validated tools should be described to assess more specifically the NEMS effects to the laryngeal muscle function.

**Conclusion:** A NEMS standarized protocol may be usefull for clinical research allowing to assess its efficacy in SD management in ICU patients.

**References**

1. Robert D. Les troubles de la déglutition postintubation et trachéotomie. Réanimation. 2004 Sep;13(6–7):417–30.
2. Clark H, Lazarus C, Arvedson J, Schooling T, Frymark T. Evidence-based systematic review: effects of neuromuscular electrical stimulation on swallowing and neural activation. Am J Speech Lang Pathol. 2009 Nov;18(4):361–75.
3. Dziewas R, Mistry S, Hamdy S, Minnerup J, Van Der Tweel I, Schäbitz W, et al. Design and implementation of Pharyngeal electrical Stimulation for early de-cannulation in TRACheotomized (PHAST-TRAC) stroke patients with neurogenic dysphagia: a prospective randomized single-blinded interventional study. Int J Stroke Off J Int Stroke Soc. 2017 Jun;12(4):430–7.
4. Malandraki GA, Markaki V, Georgopoulos VC, Psychogios L, Nanas S. Postextubation dysphagia in critical patients: a first report from the largest step-down intensive care unit in Greece. Am J Speech Lang Pathol. 2016 May 1;25(2):150–6.
5. Lee KW, Kim SB, Lee JH, Lee SJ, Ri JW, Park JG. The effect of early neuromuscular electrical stimulation therapy in acute/subacute ischemic stroke patients with dysphagia. Ann Rehabil Med. 2014 Apr;38(2):153–9.
6. Sallum RAA, Duarte AF, Cecconello I. Analytic review of dysphagia scales. Arq Bras Cir Dig ABCD Braz Arch Dig Surg. 2012 Dec;25(4):279–82.

## CO-38

### Lung ultrasound may improve the choice of chest physiotherapy treatment. A case report of pulmonary oedema

#### Daniel Ferreiro Carballal^1^, Carlos Diaz Lopez ^1^, Julie Rodriguez^1^, Maria Bonarek ^1^, Pascal Selot^1^, Andreia Lopes^1^, Stéphane Henriot^1^, Julie Marais^1^, Aymeric Le Neindre^1,2^

##### ^1^Service de rééducation, Hôpital Forcilles, Férolles-Attilly, France; ^2^UMR1231, Université de Bourgogne, Dijon, France

**Correspondence:** Daniel Ferreiro Carballa - dani_ferreiro@hotmail.com

*Annals of Intensive Care* 2018, **8(Suppl 2):**Co-38

**Introduction:** The lung ultrasound use in acute respiratory conditions may have the potential to increase the physiotherapist‘s diagnosis accuracy. This case report highlights the lung ultrasound impact on chest physiotherapy decision in patient with deteriorating respiratory conditions.

**Patients and Methods:** A 73-years-old male is referred to the Respiratory Intensive Care Unit for respiratory deterioration. The physiotherapist’s clinical examination highlights hypoxemia, tachypnea, and right thoracic side reduced mobility associated to lung auscultation crakles. Moreover, pulmonary auscultation shows crackles bilaterally. Chest X-ray reveals basal opacity and reduced intercostal spaces on the right and heart deviation to the right. We also note a bilateral and diffuse alveolar syndrome. Three clinical hypotheses may be proposed: pneumonia, pulmonary oedema or atelectasis. As the chest physiotherapy treatment may be dramatically different depending on the clinical hypothesis, the physiotherapist performs a lung ultrasound examination. The lung ultrasound shows the presence of coalescent, homogeneous and diffuse B lines, a very small pleural effusion and right diaphragm paralysis. These ultrasound findings suggest a pulmonary oedema. So, the physiotherapist chooses to implement non-invasive ventilation in seated position.

**Discussion:** The initial diagnosis retained by the physiotherapist and medical staff was pneumonia but level of uncertainty leads the physiotherapist to perform lung ultrasound. These findings have changed the therapeutic decision as treatment for pulmonary oedema is very different from pneumonia. Knowing the greater accuracy lung ultrasound have, this case report support the potential for lung ultrasound to improve the physiotherapist’s diagnosis and choice of treatment.

**Conclusion:** Lung ultrasound provides relevant information about the lung and diaphragm statuses, which may allow differential diagnosis in situations where clinical examination is limited. This may also help physiotherapists to choice the more effective chest physiotherapy technique.

## CO-40

### Reproducibility of three measurements obtained by bedside muscle ultrasonography on critically ill patients

#### Thomas Joannon^1^, Benoît Voisin ^1^, Saad Nseir^1^, François-Régis Sarhan^2^

##### ^1^Pôle de Réanimation du CHRU de Lille - Unité D, Lille, France; ^2^ CHU d’Amiens, Amiens, France

**Correspondence:** Thomas Joannon - thomas.joannon@chru-lille.fr

*Annals of Intensive Care* 2018, **8(Suppl 2):**Co-40

**Introduction:** Intensive care unit acquired weakness (ICUAW) is a frequent adverse event, associated with an increase of mortality and prolonged weaning from mechanical ventilation. Preventing ICUAW occurrence is an important part of intensive care physiotherapists activity, mainly by early mobilization (EM). Currently, early ICUAW diagnosis and EM efficiency evaluation remain difficult. Muscle measurement by ultrasound is a simple, non-invasive, non-ionizing and non-volitional assessment method at patient’s bedside, possibly utilized by physiotherapists in France since 2015. Our study focused on intra and inter-observer agreement of three measurements obtained by muscle ultrasound on critically ill patients. We hypothesized that reproducibility of measurements is independent of the qualification level of the observer.

**Patients and Methods:** Prospective observational study in our 10 beds medical ICU. Main inclusion criteria were hospitalized in ICU, age over 18 and mechanically ventilated since more than 24 h. Main non-inclusion criteria were an instability that prevented the patient from being positioned for the examination and unavailability of one of the observers. All measures were realized by B-mode ultrasonography with a 4 MHz curvilinear array transducer. Position of the patient, sites of measures, position of the probe and pressure applied on the skin were standardized. We assessed rectus femoris cross sectional area (RFCSA), quadriceps thickness (QDT) and tibialis anterior thickness (TAT). Agreement of measures was studied in two different groups, first through measures obtained by a physiotherapist (group 1) then comparing a physiotherapist with an intensivist (group 2). Data were analyzed using Wilcoxon signed rank test. Coefficient of variation (CV) and bland–Altman analysis were used to assess agreement.

**Results:** 15 patients (n = 5 and n = 10) of mean (SD) age 62 (16) and 66 (9) years old (p = 0,511), mean (SD) BMI 25 (3,5) and 28 (6) kg m^−2^ (p = 0,352) and mean (SD) IGS2 53 (26) and 52 (17) (p = 0,606) were included. In group 1 mean CV for each muscle was less than 6% and Bland–Altman analysis showed excellent agreement between examinations. In group 2 mean CV were less than 5% for QDT measurements and for TAT. Mean CV were less than 13% for RFCSA. Bland–Altman analysis showed good agreement between observers for QDT and TAT but poor agreement for RFCSA.

**Conclusion:** Results showed excellent intra-observer agreement for the physiotherapist and the intensivist. Inter-observer agreement was poor, mostly for RFCSA. Measurement protocol adjustments could allow better results.

